# Apnoeic oxygenation with high-flow oxygen for tracheal resection and reconstruction surgery

**DOI:** 10.1186/s12871-022-01610-y

**Published:** 2022-03-18

**Authors:** Nguyen Minh Ly, Ngo Van Dinh, Dinh Thi Thu Trang, Ngo Vi Hai, Tong Xuan Hung

**Affiliations:** 1 Department of Anesthesiology and Pain Medicine, 108 Military Central Hospital, No.1 Tran Hung Dao Street, Hai Ba Trung District, Ha Noi City, 100000 Vietnam; 2 Department of Thoracic surgery, 108 Military Central Hospital, Hanoi, Vietnam

**Keywords:** Tracheal resection, Reconstruction, Stenosis, Anesthesia, High-flow, Apnoeic oxygenation

## Abstract

**Background:**

Tracheal resection and reconstruction are the most effective treatments for tracheal stenosis, but the difficulties are surgery and maintaining ventilation performed on the patient’s same airway. High-flow oxygen has begun to be applied to prolong the apnoea time in the tracheal anastomosis period for tracheal resection and reconstruction. This study aims to evaluate the effectiveness of apneic conditions with high-flow oxygen as the sole method of gas exchange during anastomosis construction.

**Methods:**

A prospective study was performed on 16 patients with tracheal stenosis, with ages ranging from 19 to 70, who underwent tracheal resection and reconstruction from April 2019 to August 2020 in 108 Military Central Hospital. During the anastomosis phase using high flow oxygen of 35–40 l.min-1 delivered across the open tracheal with an endotracheal tube (ETT) at the glottis in apnoeic conditions.

**Results:**

The mean (SD) apnoea time was 20.91 (2.53) mins. Mean (SD) time anastomosis was 22.9 (2.41) mins. The saturation of oxygen was stable during all procedures at 98–100%. Arterial blood gas analysis showed mean (SD) was hypercapnia and acidosis acute respiratory after 10 mins of apnoea and 20 mins apnoea respectively. However, after 15 mins of ventilation, the parameters are ultimately returned to normal. All 16 patients were extubated early and safely at the end of the operation. There were no complications, such as bleeding, hemothorax, pneumothorax, or barotrauma.

**Conclusion:**

High-flow oxygen across the open tracheal under apnoeic conditions can provide a satisfactory gas exchange to allow tubeless anesthesia for tracheal resection and reconstruction.

## Background

Tracheal stenosis is a rare disease often caused by prolonged intubation, primary or secondary tumors, and invasive thyroid cancer. They are life-threatening if not adequately treated [1]. Tracheal resection and reconstruction are the most effective treatment, but the difficulties are surgery and maintaining ventilation performed on the patient’s same airway [2].

Depending on the severity and location of the stenosis and the type of surgical procedure, there may be a variety of choices for perioperative airway management such as a facemask, laryngeal mask airway, tracheal intubation tube, jet ventilation with the small catheter, cardiopulmonary bypass, and extracorporeal membrane oxygenation [3, 4].

The methods like intubation or using small-sized high-frequency catheters often cause a narrow surgical field and limited vision.

Recently, high-flow nasal and high-flow oxygen delivery device attached directly to a laryngeal mask or tracheal tube has begun to be applied to provide oxygen for some laryngeal surgery without intubation as well as prolonging the apnoea time in the difficult intubation [5, 6].

We also applied this method during the tracheal anastomosis period for tracheal resection and reconstruction, aiming to create an optimal surgical field without endotracheal intubation and ventilation [7].

This study aimed to evaluate the gas exchange efficiency and safety of apnoeic oxygenation with a high-flow oxygen method, used for airway management at 16 patients’ tracheal resection and reconstruction [8].

## Methods

From April 2019 to August 2020, 16 patients were diagnosed with tracheal stenosis caused by cancer invasive thyroid cancer, tracheal stenosis after intubation, and upper tracheal tumor. They were scheduled for tracheal resection and reconstruction surgery using high-flow oxygen combined for airway management at the Department of Anesthesia of 108 Military Central Hospital.

### Preoperative preparation and materials

The equipment and anesthesia agents:

- Anesthesia machine, multi-parameter monitor, TCI (target-controlled infusion) system.
- Fibroscope, rigid bronchoscope
- High-flow oxygen system
- Flexible endotracheal tubes (ETT), ID sizes from 4.0 to 7.5. The Proseal laryngeal mask size is 3–5. Catheters with diameters of 3.3–4.7 mm (Cook Airway Exchange Catheter) can pass through narrow positions for high-frequency jet ventilation.
- Anesthesia agents: Propofol, rocuronium, fentanyl, morphine.
- Emergency instruments.

Evaluation of patients:

Evaluation of patients includes general history and physical examination, with particular attention to the airway and pulmonary systems, and analyzing arterial blood gases.

To prepare for any possible airway emergency during the induction and maintenance of anesthesia, it is essential to carefully evaluate the stenosis or tumor’s exact location and the obstruction degree preoperatively.

**Fig. 1:**
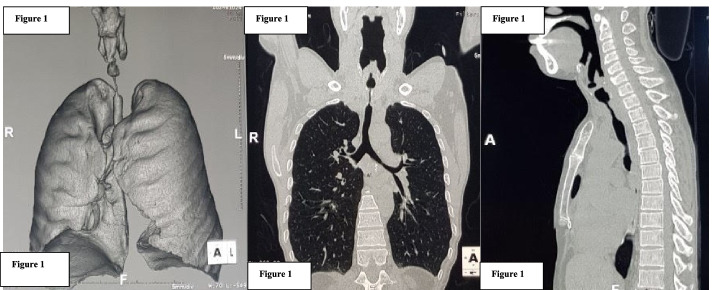
CT scan image of tracheal stenosis after intubation

Computed tomography (CT) scans provide the greatest diameter of the tracheal stenosis or tumor, minimum tracheal diameter, the length of the lesion, the distance from the vocal cord to the tracheal stenosis, from stenosis to the carina… (Fig. 1).

Bronchoscopy defines the character of the tracheal stenosis or tumor surrounding the tracheal by a direct vision that can be predicted capacity intubated and operated.

## Method

Before the induction, it must be considered that spontaneous breathing or mechanical ventilation is likely to be possible through the stenosis with general anesthesia. Nevertheless, severe airway obstruction may occur during the induction of general anesthesia, and thus the appropriate backup method will be required to prevent disaster.

The surgeon should always be in the OR during induction and available to manage a surgical airway if this becomes necessary. A rigid bronchoscope, trans tracheal jet ventilation (TTJV) must be immediately available.

If possible, mask ventilation under general anesthesia, preoxygenation with 6 l.min-1100% for 5 mins, slow and gentle induction of anesthesia followed by IV: fentanyl 2 μg/kg, propofol TCI Cp 3.5–4 μg/ml, rocuronium 0.6 mg/kg.

The positive pressure ventilation was secured via facemask, inserting the endotracheal tube into the tracheal so that the tip of the endotracheal tube is close to the stenosis if the distance from the vocal cord to the lesion >2 cm. If mild tracheal stenosis, we use tube 5.0–6.0 Fr passed through the narrow position.

If the distance from the vocal cord to the lesion is very short <2 cm, the Proseal laryngeal mask (LMAP) is placed, inserted sonde gastric through the second tube of LMAP, continuous ventilation via LMAP.

If the patient has severe stenosis and airway obstruction, we use catheters with diameters of 3.3–4.7 mm. (Cook Airway Exchange Catheter) put through narrow positions for high-frequency jet ventilation with 100% oxygen.

In case the patient has a tracheostomy, ventilating through the tube of tracheostomy.

Anesthesia was maintained intravenously by the combination of propofol using Target Controlled Infusion (TCI) method 3.5–4 μg/ml, fentanyl 2–3 μg /kg/h, rocuronium 0.2 mg/kg/h, methylprednisolone 2 mg/kg.

Once the airway is opened, the surgeon inserts a flexible endotracheal tube 6.5–7.5 Fr into the distal airway and ventilates. They were completely removing the laryngeal mask, endotracheal tube, catheter Cook. Setting a waiting endotracheal tube at glottis and connecting to the high-flow oxygen system with FiO2 100%.

**Fig. 2:**
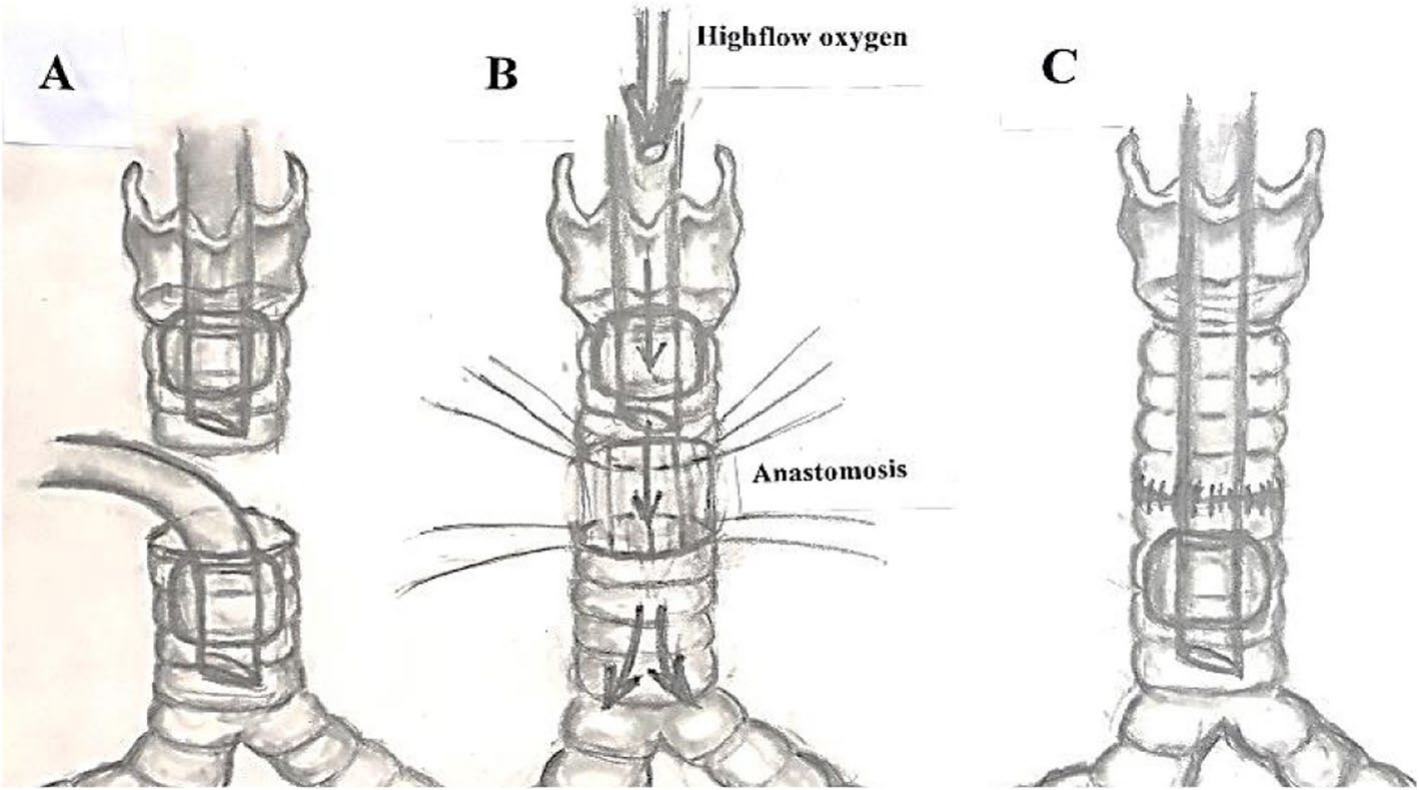
Diagram of the equipment connections*.***A**: Open airway, a flexible endotracheal tube in the distal airway. **B***.* High-flow oxygenation during anastomosis*.***C**. Anastomosis completed

**Fig. 3:**
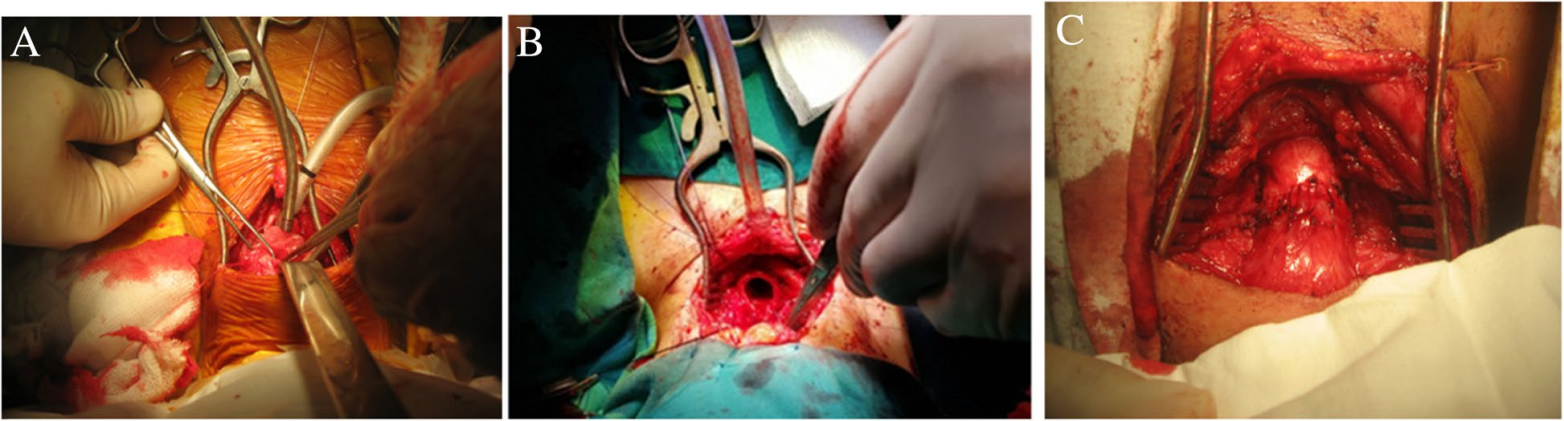
Some intraoperative images. **A**: Open airway, a flexible endotracheal tube in the distal airway. **B**. High-flow oxygenation during anastomosis. **C**. Anastomosis completed

Once the tracheal lesion is removed, and the surgeon starts anastomosis, open the oxygen flow 35–40 l.min-1 so that the oxygen is provided across the open trachea. Adjust for the direction of oxygen flow straight to the distant tracheal. During this period, the patient is still under general anesthesia, has neuromuscular blocking agents, and stops breathing completely. SpO2 and arterial blood gases were monitored. If SpO2 drops <90%, ventilation supports 100% oxygen with an endotracheal tube at the tracheal distance. When the tracheal is nearly closed, push the endotracheal tube so that the cuff passes over the anastomosis and normalizes the ventilation (Figs. 2 and 3).

End of surgery: Extubation is the primary goal because postoperative mechanical ventilation is associated with anastomotic failure; the reconstructed airway may be tenuous. When the patients were awake and cooperative, all vital parameters were normal limits; patients should be extubated on the table as soon as possible.

### Monitoring and evaluation criteria

- Characteristics of the patients: Age, height, body weight, classification of dyspnea according to MMRC (Modified Medical Research Council), degree of the physical state of the patients ASA (American Society of Anesthesiologists). Degree of tracheal stenosis according to Cotton- Mayer

- Surgical characteristics: Anesthesia time, surgery time
- The method of maintaining ventilation.
- The anastomosis time: from the tracheal open to the end closed airway.
- Apnoeic time using high flow
- The vital parameters were monitored continuously: Heart rate, SpO2, EtCO2, Blood pressure. All results are expressed as mean ± SD, blood gases analysis (ABG) at 5 periods: T0: Before the anesthesia; T1: Before using high flow. T2: After using high-flow 10 mins; T3: After using high-flow 20 mins and T4: 15 mins after high-flow finishes.

The complications in surgery include airway obstruction, tumor peeling, gas overflow, pneumothorax.

## Results

The mean age of the group was 46.50 ± 16.42 years. The proportion of males in the group (56.25%) was higher than females (43.75%). Most patients were ASA III, 02 patients were ASA IV, who have life-threatening dyspnea requiring emergency surgery (Table 1). The cause of tracheal stenosis, classification of dyspnea, and degree of tracheal stenosis were shown in Tables 2, 3 and 4. There are 11 patients classified dyspnea grade 2 and 3 according to the Modified Medical Research Council, respectively with grades 2 and 3 of tracheal stenosis according to Cotton- Mayer (Table 4). The average apnoea time was 20.91 ± 2.53 mins which means the time is enough for anastomosis. Additional information is shown in Table 5.

**Table 1:** Characteristics of the patients

Age (years)$\overline{X}$± SD Min-Max	Height(cm) $\overline{X}$± SD Min-Max	Weight(kg) $\overline{X}$± SD Min-Max	Gender		ASA		
			Male n(%)	Female n(%)	II n(%)	III n(%)	IV n(%)
46.50 ± 16.42 19–70	160.06 ± 5.16 152–168	50.50 ± 3.39 43–55	09(56.25)	07(43.75)	06(37.5)	08 (50)	02(12.5)

**Table 2:** Cause of tracheal stenosis

Causes	Number of patients (n)	Percentage %
After prolong intubaion	09	56.25
After tracheotomy	02	12.5
Tracheal tumour	01	6.25
Thyroid cancer invasion	04	25
Total	16	100

**Table 3:** Classification of dyspnea (Modified Medical Research Council)

*Dyspnea scale*	Grade 1	Grade 2	Grade 3	Grade 4	Total
n	3	6	5	2	16
%	18.75	37.5	31.25	12.5	100

**Table 4:** Degree of tracheal stenosis according to Cotton- Mayer

Degree of tracheal stenosis (%)	Number of patients	Percentage %
Grade: luminal narrowing <50%	03	18.75
Grade 2: luminal narrowing 51…. < 71%	06	37.5
Grade 3: luminal narrowing 71…. < 99%	05	31.25
Grade 4: luminal narrowing >99%	02	12.5
Total	16	100

**Table 5:** Surgical characteristics

Time (mins)	Min- Max	Value $\overline{X}$± SD
Duration of anesthesia	115–220	170.69 ± 31.89
Duration of surgery	95–185	134.56 ± 21.18
Duration of anastomosis	17–28	22.9 ± 2.41
Oxygen high-flow (apnoea time)	16–28	20.91 ± 2.53

**Fig. 4:**
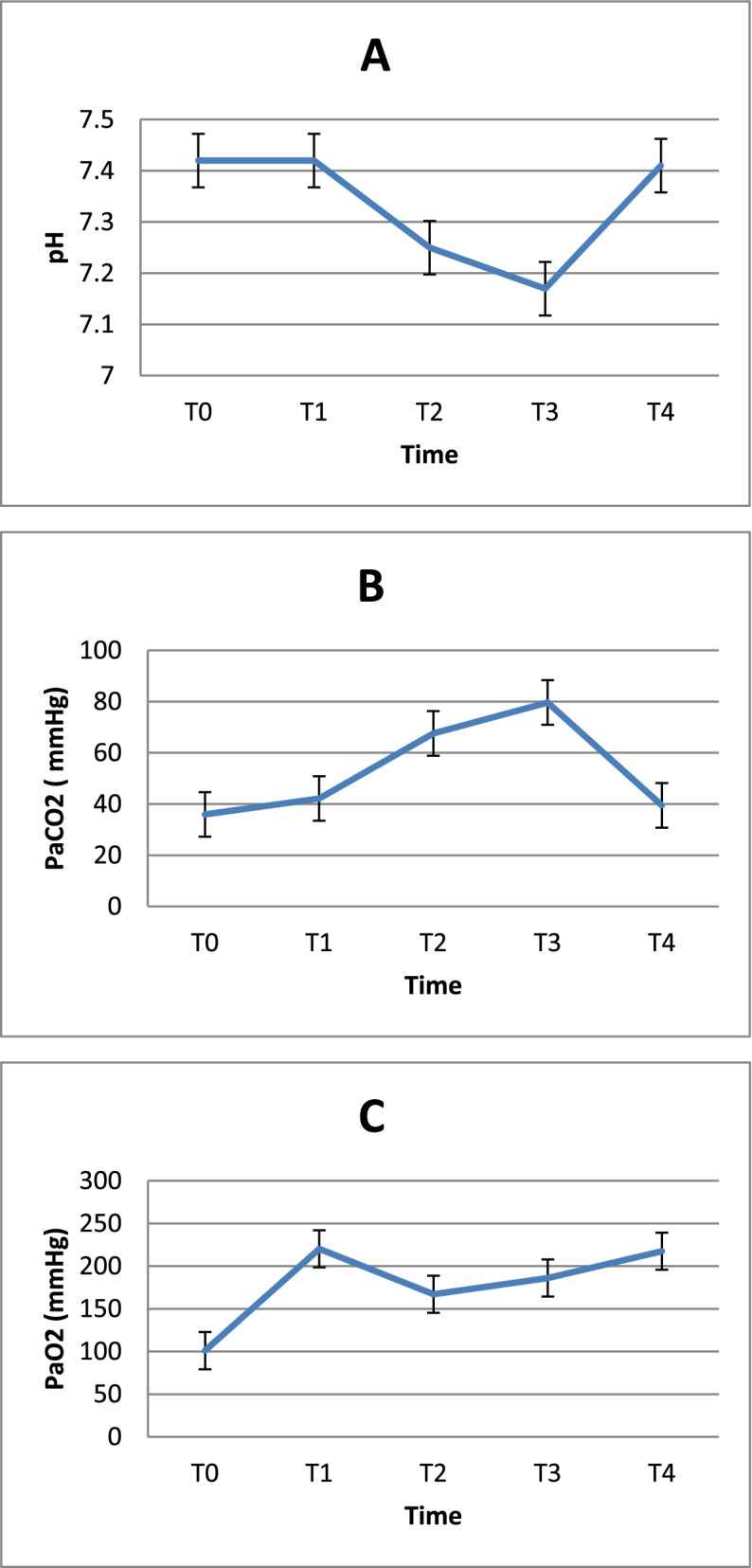
**A** pH, **B** partial pressure of arterial carbon dioxide (PaCO2) and **C** partial pressure of arterial oxygen (PaO2) at T0: Before anesthesia; T1: Before using high flow. T2: After using high flow 10 mins; T3: After 20 mins of high flow and T4: Finish the high-flow 15 mins

There may be a variety of choices for perioperative airway management. In the research, there was one patient with hypoxia who require urgent interruption of surgery must use high-flow oxygen combined ventilation intermittently through endotracheal tubes at the distant tracheal (Table 6). During the period high-flow (T2, T3), PaO2 improved significantly compared to the time of T0; acute respiratory acidosis clearly showed pH decreased, PaCO2 and HCO3- increased. However, these data return to normal at the time of T4 (Table 7 and Fig. 4). Heart rate and MAP after high-flow oxygen was decreased significantly compared with T0 (before induction), SpO2 at T0 was significantly lower than T1, T2, T3. After 20 mins of high flow (T3), etCO2 was significantly higher than before and after using high-flow oxygen (Table 8).

**Table 6:** Airway management

Period	Methods	Number of patients	Percentage %	Total
***Induction (before dissection)***	Intubation above the lesion	04	25	100%
	Intubation through the lesion	06	37.5	
	Laryngeal mask	03	18.75	
	At tracheostomy	02	12.5	
	The Small catheter for jet ventilation	01	6.25	
***Open airway (Anastomosis)***	High-flow oxygen single	15	93.75	100%
	High-flow oxygen combined ventilation intermittent *(Rescue mechanical ventilation)*	01	6.25

**Table 7:** Arterial blood gas exchange data

Time Data	T0 $\overline{X}$± SD	T1 $\overline{X}$± SD	T2 $\overline{X}$± SD	T3 $\overline{X}$± SD	T4 $\overline{X}$± SD
pH	7.42 ± 0.02	7.42 ± 0.06	7.25 ± 0.04*	7.17 ± 0.05*	7.41 ± 0.06
PaCO_2_ (mmHg)	35.95 ± 3.32	42.17 ± 9.63	67.57 ± 14.71*	79.63 ± 13.39*	39.48 ± 5.17
PaO_2_ (mmHg)	101.1 ± 3.54	220.38 ± 62.08*	167.12 ± 76.23*	186.19 ± 60.14*	217.63 ± 74.63*
Lactat	1.0 ± 0.42	1.16 ± 0,51	1.06 ± 0.6	1.06 ± 00.58	1.26 ± 0.87
HCO_3_^−^(mEq/L)	23.35 ± 3.75	27.34 ± 5.45	29.79 ± 6.73*	30.03 ± 5.5*	25.98 ± 4.78
BE (mEq/L)	2.4 ± 0.99	4.57 ± 6.32	3.23 ± 6.08	2.91 ± 5.31	2.22 ± 4.99

*: *p* < 0.05 compared with T0

**Table 8:** Change of respiratory and hemodynamic

Time Data	T0 $\overline{X}$± SD	T1 $\overline{X}$± SD	T2 $\overline{X}$± SD	T3 $\overline{X}$± SD	T4 $\overline{X}$± SD
Heart rate *(beat/min)*	87.3 ± 7.5	76.58 ± 3.42	78.5 ± 4.89	79.17 ± 6.19	77.17 ± 5.62
MAP*(mmHg)*	85 ± 12	74.5 ± 3.78	74.33 ± 5.14	75.42 ± 6,43	77.16 ± 3.76
SpO_2_ (%)	94 ± 3.7	99.83 ± 0.58*	99.17 ± 1.11*	99.67 ± 0.49*	99.66 ± 0.65*
EtCO_2_*(mmHg)*		35.67 ± 3.39		55.4 ± 7.16 ┼	33.58 ± 2.78

* *p* < 0.05 compared with T0; ┼ *p* < 0,05compared with T1

Side effects (Table 9) in surgery were acute respiratory acidosis in all 16 patients, 01 patients with hypoxia had to require interruption surgery and rescue ventilation intermittent through endotracheal tubes at the distant tracheal. There were no complications of arrhythmia, pneumothorax, hemothorax, pulmonary barotrauma.

**Table 9:** Side effects

Complications	Number of patients (n)	Percentage %
Acute respiratory distress (PaO2 < 60, SpO2 < 90)	1	6.25
Acute respiratory acidosis (PaCO2 > 50 mmHg)	16	100
Respiratory obstruction	0	0
Arrhythmia	0	0
Pneumothorax, hemothorax, pulmonary barotrauma	0	0

## Discussion

There are many treatment methods for tracheal stenosis such as conservative treatment, endoscopic treatment by luminal restoration with the main aim is to dilate the stenotic segment to match as closely as possible the normal proximal and distal diameters by using cold knife Laser: CO2, Nd-YAG, diode Diathermy Argon plasma or Cryoprobe Mechanical dilatation (dilators, rigid bronchoscopes) CRE Balloons [9–13]. Then maintenance therapy with Mitomycin, Steroids, Brachytherapy, and stents [14]. These methods offer good short-term effects and provide temporary relief, but it usually isn’t a long-term solution. In some situations, they can worsen the stenosis. Most of our patients with narrow airways were admitted to the hospital due to shortness of breath, there are 11 patients classified dyspnea grade 2 and 3 according to the Modified Medical Research Council, respectively with grades 2 and 3 of tracheal stenosis according to Cotton- Mayer. There are 03 cases of patients who can not lie, 02 cases of life-threatening who have to do the emergency surgery are classified as grade 4 dyspnea corresponding to luminal narrowing >99% (Table 3), 9 of our 16 patients had received conservative treatment but failed or had recurrent stenosis so that all patients were indicated for surgery.

These days, tracheal resection and reconstruction are known as the standard treatment for tracheal stenosis and tumor. Outcomes after tracheal surgery are usually very good, which can provide a long-lasting cure to these patients [1, 2, 14, 15].

The course of anesthesia was divided into five phases. First: induction and intubation, a critical period. Second: dissection, a period of relative calm during which lesion is defined. Third: open airway, a crucial period in which anastomosis is being constructed. Fourth: closure and emergence and fifth: extubation [2, 15, 16].

Induction is a critical period that needs to combine many flexible methods to control ventilation. The results in Table 6 show that depending on the severity and location of the stenosis, there may be various choices for airway management in the period of induction. The endotracheal tube may be placed above (25%) or through the lesion (37.5%), there are 3 patients (18.75%) showing the distance from the vocal cord to the lesion very short (< 2 cm) so we had to use a laryngeal mask (LMAP) for ventilation and found it to be a quite safe and effective solution. The LMA has also been applied to solve some of the problems posed by tracheal intubation during tracheal surgery [17–19].

J.V. Divatia et al. [18], Byung Cheul Shin et al. [19] have used the LMAP for airway management successful without any complications for surgery of a high tracheal tumor and high tracheal stenosis sited near the vocal cord, it is difficult to manage airway using a cuffed endotracheal tube. They found that the LMA has advantages when used in general anesthesia for tracheal surgery, especially with tumors situated just below the vocal cords.

Patients with severe tracheal stenosis more than 90% were under a high risk of airway obstruction. By using high-flow oxygen, the safety of these patients can be ensured. Bricker DL et al. [17]; C.L Chiu et al. [20], CHEN Hai-hong et al. [5] applied the method of cardiopulmonary bypass. This method was an easy way to ensure gas exchange. However, systemic anticoagulation theoretically increased bleeding risk, especially in the case of extensive dissection which often led to lung manipulation.

Before the induction, we prepared all emergency equipment to prevent tracheal obstruction. Small-sized catheters to pass through the narrow for jet ventilation, surgeons, as well as surgical facilities, are prepared for tracheotomy. In 2 of our patients with tracheal stenosis >90%, 01 patients can be intubated and ventilated above the narrow space. For the other, we had to use a small jet catheter pushed through the stenosis for jet ventilation with oxygen pure 100% before the open airway phase.

The open airway phase is a critical period in which anastomosis is being constructed. Once the airway is opened, a flexible endotracheal tube 6.5–7.5 Fr was inserted into the distal airway and ventilated, waiting for endotracheal tube through the glottis and connected to the high-flow oxygen system. When the surgeon started anastomosis, open the oxygen flow 35–40 l.min-1 so that the oxygen is provided across the surgical field to the distant trachea. The time for apnoeic oxygenation or the time of anastomosis was 16–28 mins, but this time depends on the surgeon’s experience. If the apnoea time is too long, which can lead to unsafety like hypercapnia or hypoxia (SpO2 < 90%, blood pH <7.1).

In the period of anastomosis being constructed 10 and 20 mins (T2, T3), with high flow oxygen 35–40 l.min-1, the blood oxygen pressure improved significantly compared to the time of T0 with PaO2 > 170 mmHg. The acute respiratory acidosis present, the lowest at T3 with pH was 7.17 ± 0.05, and PaCO2 was 79.63 ± 13.39 HCO3- increased significantly but returned to normal immediately after 15 mins mechanical ventilation at T4 (Table 7).

Tracheal resection and reconstruction require the anesthesiologist and the surgeon to share the airway. The greatest benefit of high flow is creating a free surgical field, optimal conditions for anastomosis, and no interruption of surgery without endotracheal intubation and ventilation [10].

Apnoeic oxygenation is a term that refers to a patient’s ability to receive oxygen in the absence of pulmonary movement. At the beginning of apnoea, oxygen is continuously transferred from the alveolus to the circulation to meet the body’s metabolic demands. This oxygen transfer results in the alveoli being emptied and the alveolar pressure decreasing, which is initially compensated for by alveolar volume loss due to elastic recoil and carbon dioxide transport from the blood to the alveolus. These compensatory systems rapidly deplete, and for oxygenation, a pressure gradient between the upper airway and the alveolus emerges [9, 13, 21].

M Egan et al. also applied 100% oxygen at a flow rate of 40 l.min-1 delivered across an open trachea in a case of an apnoeic female patient with subglottic tracheal stenosis, which resulted in 42-min uninterrupted surgery. The oxygen saturation rate remained more than 96% during the apnoeic period. Moreover, arterial blood gas parameters were within acceptable limits. There was no urgent interruption of surgery or rescue mechanical ventilation [10].

The investigation was conducted between November 2016 and May 2017 by C. Lyons; M. Callaghan et al. During apnea with high-flow nasal oxygen, 28 patients underwent tubeless laryngeal or tracheal surgery. Apnoea lasted a median of 19 (15–24) minutes. Four patients experienced a brief period of oxygen deficiency between 85 and 90%. At baseline, the carbon dioxide partial pressure (PaCO2) was 6.29 (0.71 kPa), and after 15 min of apnoea, it was 9.44 (1.12) kPa. The authors found that high-flow nasal oxygen provided during apnea may be sufficient for tubeless anesthesia for laryngeal surgery [9].

The safe breathing time was calculated from the time when the patient stopped breathing until the SaO2 ratio was less than 90%. Oxygen kept being exchanged in the alveoli even when there were no diaphragmatic movements or lung expansion. In the cases of healthy patients under apnoeic conditions, there were about 200–250 ml /min of oxygen move from the alveoli into the bloodstream. There were only 8–20 ml/min of carbon dioxide moved into the alveoli during apnoea. The rest of the carbon dioxide was buffered in the bloodstream because of its high water solubility. In the cases of healthy patients under ideal conditions, the PaO2 ration could be maintained more than 100 mmHg for up to 100 min without a breath. However, poor ventilation would significantly cause hypercapnia and acidosis. Many authors had demonstrated that acute respiratory acidosis within pH > 7.15 was the acceptable safety limit in case of no contraindications [10, 21, 22].

The results of Tables 8 and 9 showed that during procedure hemodynamics, oxygen pressure, oxygen saturation (SpO2) were within normal limits. Only 1 patient with hypoxia during surgery is the case of patients with pneumonia in the right lower lobe should support oxygen through the distant airway.

High-flow oxygen under apnoeic conditions can provide a satisfactory gas exchange with oxygenation indices improved than before surgery. The surgical field is especially completely spacious, with optimal conditions for anastomosis, and no interruption of operation without endotracheal intubation.

## Conclusion

In the tracheal resection and reconstruction, high-flow oxygen through the open tracheal under apnoeic conditions can provide a significant gas exchange for tubeless anesthesia. The surgical field is completely spacious and under optimal conditions for anastomosis. There were no complications of arrhythmia, pneumothorax, hemothorax, and pulmonary barotrauma.

### Limitation of the study

The limitation of this study there is the lack of transcutaneous Carbon Dioxide Monitoring (TcCO2), continuously monitoring of CO2 may allow prevention and early detection of the risk of hypercapnia at dangerous levels. In addition, the number of patients in the study group is not large enough.

## Data Availability

The datasets generated and analyzed during the current study are available from the corresponding author on reasonable request.

## Abbreviations

ETT: Endotracheal tube
TCI: Target-controlled infusion
TTJV: Transtracheal jet ventilation
LMAP: Laryngeal mask Proseal
MMRC: Modified Medical Research Council
ASA: American Society of Anesthesiologists
